# Upper abdominal shape as a risk factor of extended operation time and severe postoperative complications in HCC hepatectomy through subcostal incision

**DOI:** 10.1186/s12957-015-0702-7

**Published:** 2015-10-13

**Authors:** Yi-fu Hou, Yong-gang Wei, Bo Li, Jia-yin Yang, Tian-fu Wen, Ming-qing Xu, Lv-nan Yan, Wen-tao Wang

**Affiliations:** Department of Hepatic Surgery, West China Hospital, Sichuan University, Chengdu, 610041 Sichuan Province China

**Keywords:** Upper abdominal shape, Hepatocellular carcinoma, Open surgery, Postoperative complications

## Abstract

**Background:**

Subcostal incision is the most widely used approach in open surgery for patients with hepatocellular carcinoma (HCC). Body shape is recognised to be a factor influencing the difficulty of surgery; however, the exact impact of the increased difficulty on the patients’ operation as well as the outcome has not been analysed. In this study, we retrospectively studied the possible influence of patients’ body shape, tumour burden and varied surgical methods on the operation procedure and postoperative complications.

**Methods:**

From January 2009 to December 2013, 651 patients with HCC were included in the study. We studied the patients’ sex, age, body mass index, upper abdominal body shape described by the depth-to-width ratio for the trunk at the celiac axis on CT/MRI, Child-Pugh classification, tumour burden and a different liver dissection method before the surgery and used a regression model for analysis.

**Results:**

Prolonged operation time is associated with advanced tumour stage, large CA ratio, previous abdominal surgery, selective hepatic vascular occlusion and dissecting with Cavitron ultrasonic surgical aspirator rather than clamp crushing. Surgical blood loss is associated with operation time, liver function and a different liver dissection method. The incidence of severe postoperative complication was 17.5 % (114/651) and was associated with larger CA ratio, Child-Pugh stage B liver function and greater blood loss.

**Conclusions:**

Large upper abdominal shape is a risk factor of both prolonged operation time and severe postoperative complication. CA ratio combined with liver function and surgical blood loss has an acceptable power to predict severe postoperative complications.

## Background

Liver resection is a potential curative treatment for hepatocellular carcinoma (HCC) along with liver transplantation [1]. Subcostal incision is the conventional approach in open surgery for hepatectomy [2]. Other incisions, such as upper midline incision (UMI) with or without laparoscopic assistance, have been proven to be safe and feasible by some studies [3, 4]. Kwang et al. reported that larger upper abdominal shape was associated with extended operation time in transplantation donors using UMI. Moreover, some studies reported that a larger body shape can be the risk factor for some postoperative complications [5]. However, in subcostal incision settings, a few studies have discussed the exact impact of the increased difficulty on patients’ operation as well as the outcome.

This retrospective study was designed to investigate the questions mentioned above.

## Methods

### Patients

From January 2009 to December 2013, we reviewed 788 patients who underwent hepatectomy through a subcostal incision in West China Hospital of Sichuan University. Of the total, 137 patients were excluded because preoperative CT/MRI scans were not available, and the remaining 651 patients were included in the study. All the patients were preoperatively diagnosed with HCC according to AASLD criteria 2010 edition, and a postoperative pathological examination confirmed the diagnoses [6]. This study was approved by the institutional review board of Sichuan University.

### Preoperative conditions

All CT or MRI scans were obtained within 2 weeks before operations, and we calculated the depth-to-width ratio for the trunk at the celiac axis (CA) on from the scans. This CA depth ratio represented the upper abdominal shape of the patients [5]. Child-Pugh classification was used to measure the patients’ liver function. We searched the patients’ history of abdominal surgery, such as cholecystectomy, appendectomy, splenectomy, and accompanied chronic diseases, such as hypertension, diabetes and chronic obstructive pulmonary disease (COPD), through the medical records department of the hospital. The TNM staging system from the AJCC cancer staging manual 7th edition was applied to assess the tumour burden [7].

### Surgical methods and technique

The right subcostal incision with a medial extension to the xyphoid process is approximately 20–40 cm in length according to the patients’ different body shapes [2]. We used the Cavitron ultrasonic surgical aspirator (CUSA) or clamp crushing as the two main methods for parenchymal transaction. For different vascular occlusion options, we divided the patients into two groups, continuous/intermittent selective hepatic vascular occlusion and no vascular occlusion. After the completion of resection, all patients had surgicel and fibrin sealant to prevent further bleeding. There were two groups of experienced general surgeons that separately completed all of the 651 hepatectomies.

### Operation time and blood loss

We considered operation time longer than 220 min as a prolonged operation time. We divided surgical blood loss at a cut-off point of 500 ml. These two indicators can generally assess the difficulty of a surgery.

### Postoperative complication

Mild ascites were the most common complication after liver resection [8], which is a grade II complication according to the Clavien classification [9]. Using a peritoneal cavity drainage tube and intravenous diuretics and albumin, it was usually easily controllable [10]. In this study, we considered postoperative pneumonia and any grade III to grade V complications, which prolonged patients’ recovery time significantly, as a severe postoperative complication. These included postoperative bleeding, re-operation, shock, postoperative death and postoperative pneumonia, as mentioned above.

### Statistical analysis

To identify the possible risk factors of prolonged operation time, we applied Student’s *t* test in a univariate analysis and a forward step-wise multiple linear regression model in multivariate analysis. A logistic regression was applied to analyse the risk factors for greater amounts of surgical blood loss. Fisher’s exact test and chi-square test were used to compare the incidence of severe postoperative complication in univariate analysis, and a logistic regression analysis model was applied in the multivariate analysis. A receiver operating characteristic (ROC) curve was used to predict the incidence of severe postoperative complication. All analyses were performed with SPSS version 21.0 (IBM SPSS Inc., Chicago, IL). A *P* value <0.05 was considered statistically significant.

## Results

### Patients’ physical condition and tumour burden

Of the 651 HCC patients, 347 patients underwent right lobe hepatectomies, 159 patients underwent left lobe hepatectomies, and 145 patients underwent middle segment hepatectomies. The component ratio of right/middle/left hepatectomy in the larger CA group (185/60/60) is not statistically significantly different to the smaller CA group (174/87/85; *P* = 0.678). The physical condition and tumour burden of all 651 patients are summarised in Table 1. The mean age of the patients was 51.2 years (range 12–78 years), the mean CA depth rate was 0.36 and there were 549 male (84.3 %) and 102 female (15.7 %). The mean body mass index (BMI) was 23.4, and 83 out of 568 patients had a history of abdominal surgery, such as appendectomy, cholecystectomy, splenectomy and previous HCC resection. Accompanied chronic diseases, such as hypertension, COPD, diabetes, tuberculosis and chronic kidney disease, were found in 164 patients. According to the Child-Pugh classification, 615 patients (94.5 %) had a grade A liver function and 36 patients (5.5 %) had a grade B liver function. Tumour burden was quantified by TNM staging system from the AJCC cancer staging manual 7th edition, and there were 490 patients (75.3 %) within stage II and the remaining 161 were in advanced stage (24.7 %).

**Table 1:** The patients’ baseline surgical information

Factors	Value
Physical condition and tumour burden	
Age (years)	51.2 ± 11.9 (12–78)
Male sex [*n* (%)]	549 (84.3)
Weight (kg)	62.2 ± 9.2 (37–115)
BMI	23.4 ± 3.2 (16.8–30)
CA depth ratio	0.36 ± 0.05 (15.39–35.39)
Previous abdominal surgery [*n* (%)]	83 (12.7 %)
Accompanied chronic disease [*n* (%)]	164 (25.2 %)
Child-Pugh classification A grade [*n* (%)]	615 (94.5 %)
TNM stage I and II [*n* (%)]	490 (75.3 %)
Surgical methods and techniques	
Operation time (minutes)	216.8 ± 38.3 (145–400)
Blood loss (ml)	424.4 ± 347.0 (10–3000)
Selective hepatic vascular occlusion [*n* (%)]	430 (66.1 %)
CUSA [*n* (%)]	463 (71.1 %)
Surgeon group A [*n* (%)]	352 (54.1 %)

### Operation method and technique

The operation methods and techniques were also summarised in Table 1. The mean operation time was 216 min (range 145–400 min). The median blood loss was 340 ml (range 10–3000 ml). There were 213 (32.6 %) cases of ≥500 ml surgical blood loss. Selective hepatic vascular occlusion manoeuvre was applied in 430 cases (66.1 %). In 463 cases (71.1 %), CUSA was used as a main transaction tool, and in the rest of the 188 cases (28.9 %), we used clamp crushing. Two groups of surgeon completed all 651 cases of hepatectomies, with group A finishing 352 cases (54.1 %) and group B finishing 299 cases (45.9 %).

### Postoperative complications and hospitalization

Five hundred and thirty-seven patients (82.5 %) had mild ascites after resection, 144 patients (17.5 %) suffered from severe complications, six patients (0.9 %) died, and among them, four patients died of liver failure after the surgeries and two patients died of the postoperative abdominal haemorrhage. The median hospital stay was 9 days (range 3–66 days) and the median ICU stay was 1 day (range 0–11 days). For those patients who suffered from severe postoperative complication, the best medical care was approached in time.

### Correlation of prolonged operation and possible risk factors

In univariate analysis, advanced tumour stage (*P* < 0.001), larger upper abdominal body (CA > 0.36, *P* < 0.001), previous abdominal surgery (*P* < 0.001), selective hepatic vascular occlusion (*P* < 0.001) and use of CUSA (*P* < 0.001) were significantly associated with prolonged operation time. In multiple linear regression of multivariate analysis, we had the same result, with a determinate coefficient *R*^2^ = 0.224 (Table 2).

**Table 2:** Risk factors associated with prolonged operation time in 651 HCC patients

Multivariate analysis		
Possible risk factors	*B* (95 % CI)	*P* value
Age	–	NS
≤50 years = 0		
>50 years = 1		
Sex	–	NS
Female = 0		
Male = 1		
BMI	–	NS
≤24 = 0		
>24 = 1		
CA depth ratio	14.2 (8.8–19.7)	<0.001
≤0.36 = 0		
>0.36 = 1		
Previous abdominal surgery	15.8 (7.9–19.7)	<0.001
No = 0		
Yes = 1		
Child-Pugh classification	–	NS
Grade A = 0		
Grade B = 1		
TNM stage	27.2 (21.0–33.5)	<0.001
Stage I and II = 0		
Advanced stage = 1		
Selective hepatic vessel occlusion	8.5 (2.8–14.1)	0.004
No = 0		
Yes = 1		
CUSA	8.7 (2.9–14.6)	0.004
No = 0		
Yes = 1		
Determinate coefficient *R* ^2^ = 0.224		

### Logistical regression model for greater surgical blood loss and severe postoperative complications

In multivariate logistical regression, prolonged operation time (*P* < 0.001, OR = 2.40; 95 % CI, 1.66–3.47) and Child-Pugh stage B liver function (*P* = 0.023, OR = 2.33; 95 % CI, 1.12–4.83) were associated with greater surgical blood loss. The use of CUSA rather than clamp crushing was associated with less blood loss (*P* = 0.008, OR = 0.59; 95 % CI, 0.40–0.87).

In univariate analysis, Child-Pugh B classification, CA depth ratio (CA > 0.36), history of abdominal surgery, more than 500 ml of blood loss and prolonged operation time (≥220 min) were significantly associated with severe postoperative complication. In multivariate logistical regression model, CA depth ratio, Child-Pugh B classification and more than 500 ml of blood loss were significant risk factors relating to severe postoperative complication (Table 3).

**Table 3:** Incidence of severe postoperative complication

Univariate analysis			
Possible risk factor	Severe postoperative complication cases		*P* value
CA depth ratio, ≤0.36/>0.36	45/334	69/317	0.007
History of abdominal surgery, no/yes	82/487	32/164	<0.001
Child-Pugh classification, A/B	101/615	13/23	0.006
Blood loss, <500 ml/≥500 ml	58/400	56/211	<0.001
Operation time, <220 min/≥220 min	50/361	64/290	0.007
Logistic regression in multivariate analysis			
Risk factor	Odds ratio (95 % CI)		*P* value
CA depth ratio, ≤0.36/>0.36	1.7 (1.1–2.7)		0.013
Child-Pugh classification, A/B	2.9 (1.4–6.3)		0.004
Blood loss, <500 ml/≥500 ml	2.1 (1.4–3.3)		0.001

### ROC curve in analysing the factors predicting severe postoperative complications

**Fig. 1:**
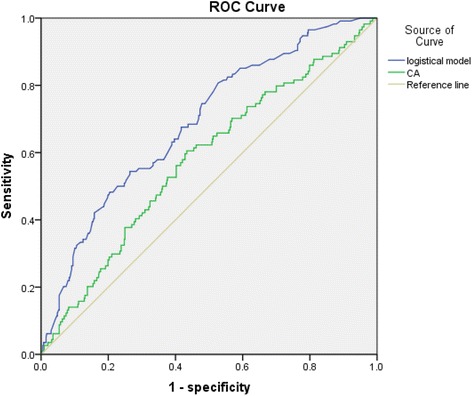
ROC curve analysis of CA ratio and logistical model for severe postoperative complication. The optimal sensitivity of 60.5 % and specificity of 62.8 % with an AUC value of 0.69

We compared CA ratio alone with our logistic regression model by using a ROC curve. CA ratio alone had an optimal sensitivity of 60.5 % and specificity of 56.6 % with an AUC value of 0.58, while our logistical regression model had an optimal sensitivity of 60.5 % and specificity of 62.8 % with an AUC value of 0.69 (Fig. 1).

## Discussion

Although it makes common sense that a larger thoracic cavity can certainly cause some difficulties in liver resection surgeries, it is the first time to identify this independent risk factor. By measuring the duration of operation time and the severity of postoperative complication, we had confirmation along with other risk factors.

Our study showed that larger CA depth ratio (CA > 0.36) together with advanced TNM stage, a history of abdominal surgery, use of hepatic vessel occlusion manoeuvre and dissection with CUSA are independent risk factors of longer operation time in HCC patients who underwent hepatectomy through a subcostal incision. Furthermore, our logistical regression model showed larger CA depth ratio (CA > 0.36) is also associated with severe postoperative complications together with a Child grade B liver function and more than 500 ml of blood loss during the surgery. This model has an acceptable power to predict severe postoperative complications in ROC curve analysis. Since it is easy to obtain the patients’ CA depth ratio through the preoperative CT scans, we believe it is necessary to measure CA depth ratio so that we can have a good understanding on operation plan and postoperative complication management. However, CA ratio was not a significant risk factor of greater surgical blood loss.

Our study was consistent with earlier reports that larger CA ratio was associated with longer operation time and some severe postoperative complications [3, 5]. Although we were not talking about the exact same operation or surgical procedure, it was clear that humans’ body shapes had certain influence on surgical procedure in different ways, and we should pay more attention to it. One of our earlier studies had discussed the influence of patients’ weight on hepatectomy. The results showed obesity was not a risk factor for postoperative complications. This study mainly focused on the patients’ bone structures, specifically the thorax shapes. Still, we had the same conclusion on BMI [11]. One thing important to explain is that we did not compare the hospitalisation status in this paper because some patients were transferred to a local rehabilitation clinic and that part of the medical record was not available.

Our study also showed some interesting findings. Selective hepatic vessel occlusion manoeuvre was widely used in hepatectomy. Theoretically, it could stop the blood inflow and made the dissection process easier. However, in our study, this surgical manoeuvre cost even more operation time. One way to explain the phenomenon is that in other cases, this manoeuvre was not required; rather, it was simply easier to complete resection in those cases. A recent study focusing on how hepatic vascular occlusion affects surgical procedures and outcomes carried out by our group showed some similar results [12]. Furthermore, CUSA required more operation time than clamp crushing but had less surgical blood loss. The reason for this might be that CUSA could complete a fine cutting edge and this delicate process was more time consuming but caused less vessel damage.

The Clavien classification of complication was the most popular system to evaluate postoperative complications. Our data showed the postoperative mortality of 0.9 %, which demonstrated that liver hepatectomy was a relatively safe procedure. In our study, we considered grade III to grade V along with postoperative pneumonia to be severe complications. We defined postoperative pneumonia as bronchovascular shadows and pulmonary consolidation seen on chest X-ray, with clinical symptoms of pulmonary rales, fever and cough. One thing that needs to be mentioned was that sputum excretion for patients with a 20- to 40-cm long incision on the right upper quadrant of the abdominal wall was relatively hard. In our experience, postoperative pneumonia significantly delays the patients’ recovery time. Larger CA depth ratios (CA > 0.36) mean longer incisions and greater trauma, which could make the incidence of severe postoperative complication higher. Child-Pugh classification grade B liver function was another important risk factor. Poor hepatic reserve meant even worse remnant liver function after resection, which would very likely lead to serious postoperative complication. Blood loss was a comprehensive indicator of surgical trauma, which could also cause lethal consequence after the surgery. In our logistic regression model of ROC curve analysis, with an AUC value of 0.69, its power to predict severe complication was not excellent but acceptable overall.

It was surprising to find that, age, sex and BMI were not risk factors for longer operation time and severe postoperative complication. There could also be some selective bias here because our surgeon would neither operate on patients of old age or bad general condition.

### Limitations

Our data comes from a single hospital. Although our hospital used to be the largest single hospital in the world, with over 8000 beds and receiving patients form all over the country, it would be better if it was a multi-centre study. Unlike hemihepatectomy in living donor liver transplantation, HCC hepatectomy must take the tumour characteristics into consideration, and it does not have a fixed operation pattern, so the heterogeneity of this study is greater than operations for living donor liver transplantation.

## Conclusions

We can draw the conclusion that, in our study, larger CA depth ratio (CA > 0.36) was an individual risk factor of longer operation time and severe postoperative complications in hepatectomy through a J-shaped incision. Since perioperative information on patients’ CA depth ratio was easy to obtain, this effortless work could give us some useful information to develop our operation plan and set precaution on patients with large upper abdominal body.
